# A roadmap towards personalized immunology

**DOI:** 10.1038/s41540-017-0045-9

**Published:** 2018-02-06

**Authors:** Sylvie Delhalle, Sebastian F. N. Bode, Rudi Balling, Markus Ollert, Feng Q. HeFeng

**Affiliations:** 1 Department of Infection and Immunity, Luxembourg Institute of Health (LIH), 29, rue Henri Koch, 4354 Esch-sur-Alzette, Luxembourg; 2 Center for Pediatrics—Department of General Pediatrics, Adolescent Medicine, and Neonatology, Medical Center, Faculty of Medicine, University of Freiburg, Mathildenstrasse 1, 79106 Freiburg, Germany; 3 Luxembourg Centre for Systems Biomedicine (LCSB), University of Luxembourg, Campus Belval, 6, Avenue du Swing, 4367 Belvaux, Luxembourg; 4 Department of Dermatology and Allergy Center, Odense Research Center for Anaphylaxis (ORCA), University of Southern Denmark, 5000 Odense C, Denmark

**Keywords:** Immunology, Systems biology, Biomarkers

## Abstract

Big data generation and computational processing will enable medicine to evolve from a “one-size-fits-all” approach to precise patient stratification and treatment. Significant achievements using “Omics” data have been made especially in personalized oncology. However, immune cells relative to tumor cells show a much higher degree of complexity in heterogeneity, dynamics, memory-capability, plasticity and “social” interactions. There is still a long way ahead on translating our capability to identify potentially targetable personalized biomarkers into effective personalized therapy in immune-centralized diseases. Here, we discuss the recent advances and successful applications in “Omics” data utilization and network analysis on patients’ samples of clinical trials and studies, as well as the major challenges and strategies towards personalized stratification and treatment for infectious or non-communicable inflammatory diseases such as autoimmune diseases or allergies. We provide a roadmap and highlight experimental, clinical, computational analysis, data management, ethical and regulatory issues to accelerate the implementation of personalized immunology.

## Introduction

Continuous improvements in laboratory technologies and computational biomedicine have enabled the generation and processing of vast amounts of data, prerequisites that will allow medicine to evolve from a “one-size-fits-all” approach to a more detailed patient stratification and future personalized treatment. With the publication of the epochal report on Precision Medicine Initiative in 2011^1^ and USA President Obama’s announcement in his state of the Union Address 2015 (https://obamawhitehouse.archives.gov/the-press-office/2015), precision/personalized medicine is becoming one of the theme songs in biomedical research across the world. Traditional approaches based on clinical symptoms and a few classic laboratory markers can only provide incomplete information on disease manifestations. Furthermore, molecular and clinical heterogeneity among patients is very common in many diseases, especially in multi-factorial complex diseases. For instance, the immune responses following the same treatments may be even individual-specific.^2–4^ As a clinical consequence, some routinely used drugs, for example statins, widely prescribed to lower cholesterol, can be beneficial only to a small fraction of patients while other drugs might even be harmful to certain ethnic groups.^5^ Therefore, it is essential for researchers and clinicians to identify the molecular and environmental factors that determine whether and how an individual patient responds to a particular therapy.

The move to personalized treatment first requires the large-scale unbiased analysis of genomic and molecular characteristics of individuals experiencing defined disease conditions to identify reliable patient-specific biomarkers linking genotypes, molecular profiles/endotypes, disease progression and “Omics” data and to process them computationally to identify personalized biomarkers.^6–11^ It is worthy to note that so far one of the most ambitious personalized medicine trials (NCT02465060, known as NCI-MATCH, launched in 2015) is recruiting thousands of participants to differentially treat individual patients suffering from solid tumors or lymphomas according to their genetic abnormalities with one of the 23 selected drugs. In 2017, two studies described therapeutic approaches using personalized vaccination targeting patient-specific mutated tumor neoantigens that gave very promising results, rising high expectations and hopes with regards to personalized medicine, at least in cancer.^12,13^ Interestingly, these recent accomplishments mainly lay on establishing effective anti-tumor immunity. Therefore, it is possible to develop personalized immunology that requires not only to test for personal genetic markers, but to identify the downstream targetable functional molecular markers and to sequentially stratify patients using multi-levels of “Omics” approaches, which shall be further advanced in personalized oncology as well as immune-centralized disease studies.

## The need of personalized immunology

Although the concept of personalized medicine in general has been proposed for a while in the field,^11^ its main successful applications are obtained in the field of cancer.

The unique feature of immune cells compared with other cell types (e.g., tumor cells) in the human body is their capability to shift between multiple activation states even under physiological conditions, not to mention under pathological conditions. The immune cells can at least switch between two states, resting and stimulated states if not counting the often-existing in-between gray areas or continuum zones. Our immune system is further complicated by many other states, including but not limited to, immatured/matured, exhausted, “anergic”, senescent and many others. These features render immunology a special layer of complexity and further increase the difficulties in delineating the underlying dynamic networks determining immune response patterns and regulating their variances among individuals. The enormous complexity of immune systems has badly called for the application of systems biology/medicine to immunology. The essential focus of systems biology is to study the emerging properties of various layers of molecular, cellular and ecological networks, instead of reductionism-based single components out of intertwined cellular and molecular networks. Therefore, the emerging properties of immune systems for the sake of the excessive existence of inter-cellular and intra-cellular immune networks^14^ cannot be revealed without the proper involvement of and further development of systems biomedicine that has already been successfully demonstrated in various related aspects of oncology. Therefore, we need to systematically characterize and profile hundreds of different immune subpopulations in each individual patient, which is the focus of systems immunology.^15^

**Fig. 1:**
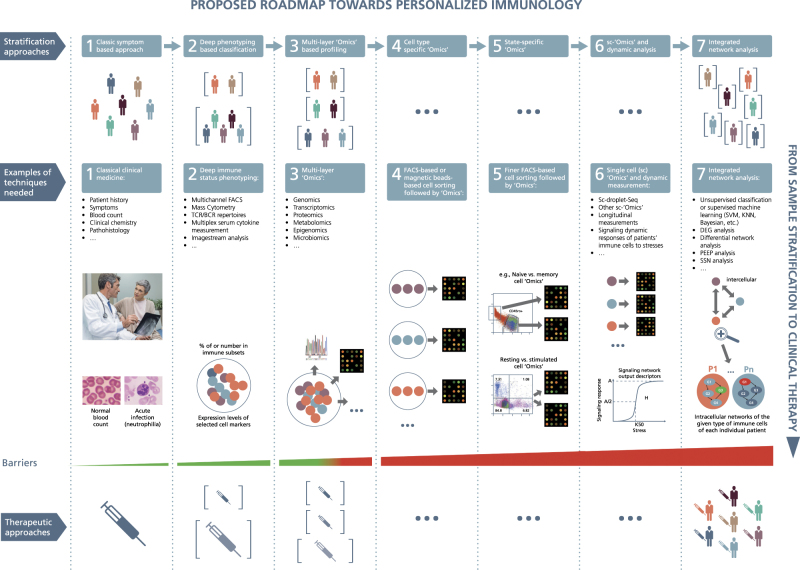
A roadmap proposed towards personalized immunology. There exist both horizontal and vertical roadmaps towards personalized immunology. Vertically, to translate sample stratification to clinical therapies, we need to utilize the state-of-the-art “Omics” analysis and network integration approaches to stratify patients into subgroups and then implement personalized therapeutic approaches to treat individual patients, which needs to overcome various types of barriers at different steps. Horizontally, we might need to go through at least 7 steps to enable personalized immunotherapies, 1) classic symptom-based approach, 2) deep phenotyping approach, 3) multi-layer “Omics”-based profiling, 4) cell type-specific “Omics”, 5) state-specific “Omics”, 6) single-cell (sc) “Omics” and dynamic response analysis of immune cells, 7) integrated network analysis. FACS, fluorescence activated cell sorting; TCR/BCR, T cell receptor/B-cell receptor; DEG, differential expression gene; PEEP, personalized expression perturbation profile; SSN, sample-specific network; SVM, support vector machine; KNN, K-nearest neighbors; under the first layer (the so-called stratification layer), different colors of patients indicate individual patients with different cellular and/or molecular profiles while brackets represent patient subgroups; under the second layer (the so-called technique layers), different small circles with distinct colors indicate different immune cells while big circles represent patient (sub)groups; under the technique layers, the snapshot of microarray representing either microarray-based or RNA-seq-based transcriptome analysis; under the third layer (the so-called therapeutic layer), the syringes with different colors or tonalities indicate different therapeutic approaches; P1,..., Pn at step 7 designate different patients; G1, G2, G3, G4 represent different genes, the arrows between them representing regulatory relationships. Three images in the second layer of step 1 are used with permissions from Fotolia.com

Furthermore, compared with tumor cells, the memory capability of our immune systems adds another layer of complexity to the particular characteristics of personalized immunology. Indeed, the responses of our immune systems are determined not only by genetic factors, but also by environmental elements. The latter such as exposed antigens will definitely affect functional hysteresis of immune cells. The complex immune-related diseases often display a mixture of various clinical symptoms and traditionally, physicians mainly divide patients into subgroups with one single disease based on their symptoms or in combination with some blood markers (Fig. 1, Step 1). Due to the subjective judgment on symptoms, physicians cannot easily and accurately classify patients based on clinical symptoms alone. To be able to more precisely stratify patients for precision and personalized treatment, there exists a strong need to identify reliable molecular biomarkers. With the development of various types of high throughput techniques, we now possess approaches that not only systematically measure frequencies of different immune subpopulations and levels of various combinations of activation/inhibitory markers, but also measure genome-scale small and macro-molecules from whole tissues to cellular levels. For instance, we can now perform immune subset deep-phenotyping (e.g., with mass cytometry/CytoF), T-cell receptor/B-cell receptor (TCR/BCR) repertoire sequencing, genome sequencing, microarray/RNA-sequencing, proteomics, metabolomics, epigenomics, microbiomics and other large-scale analyzes. Thus, we currently have opportunities to obtain high-dimensional data which are much more information-enriched and could be used as basis for biomarker discoveries and patient stratification.

The next generation of systems immunology is personalized immunology that not only applies systems biological approaches to investigate basic, translational and clinical immunology, but also aims to identify personalized biomarkers based on multi-layer chronological “Omics” and clinical data, to more precisely stratify immune-related diseases and sequentially personalize treatments (Fig. 1, Step 1–3). From the disease point of view, personalized immunology focuses on inflammatory, infectious, autoimmune, allergic and other immune-related diseases, which are apparently out of the main scope of personalized oncology. In short, we need to especially raise and develop personalized immunology, not only due to the extreme high degree of complexity in immune systems, but also attributable to the completely distinct diseases. We here elaborate on some examples of immune-related diseases such as infectious diseases, autoimmune diseases and allergy that tempt to either identify novel biomarkers, or stratify patients, or a combination of aforementioned purposes.

## Selected examples in precision/personalized immunology

Related to the terms “systems medicine” or “systems biology”, there are only a few studies registered at the major clinical trial database (https://clinicaltrials.gov/). As expected, not that many concern “precision”/“personalized” AND “immunology”/“inflammation” (only around 200). Even among those, the majority is still related to the field of oncology. In Table 1 a non-exhaustive exemplary list of trials with precision immunological aspects can be found (HIV was excluded as it is not the focus of this review). Notably, only a few of these listed immunology-disease studies actually focus on measuring “Omics” data, while many others still only investigate single aspects of a disease, or in a better scenario a combination of several particular facets that do not clearly specify a genome-scale assessment, indicating again the high demand for further development of personalized/precision immunology.

**Table 1:** Selected examples of clinical trials from ClinicalTrials.gov related to personalized/precision immunology

Clinical trial number	Disease studied	Type of samples analyzed	What is measured/planned	Status	References or comments
NCT02437084	Diabetes type 2	Blood	“Integrated omics profile” ^a^, glucose tolerance (OGTT), LDL, triglycerides	Active, recruiting	
NCT02654704	Pneumococcal vaccination	Blood	RNA-expression, protein profiles and small molecule profiles on immune cells as these change over time prior to and following immune activation by the vaccine -- >integrated omics profile	Active, recruiting	
NCT02183818	COPD	Not specified, probably blood	Omic data sets including genetic, epigenetic (methylation), gene expression, microRNA and metabolomic levels	Active, recruiting	
NCT02931955	Insect venom and pollen allergy	Blood, stool	Time-series transcriptome of various sorted CD4+ subsets, serum cytokines, PBMC immune cells deep phenotyping	Active, recruiting	
NCT00897715	Inflammation in Chronic Kidney Disease and Cardiovascular Disease	Not specified, probably blood	Polymorphism/haplotypes, genotype combinations and gene-environmental interactions that can affect inflammation	Completed	No publication found
NCT01423461	Childhood wheeze and asthma	Not specified, probably blood	“Genetic data”	Completed	No publication found
NCT01681732	Pediatric asthma	Saliva, probably blood, lung function tests	“Genetic tests”, lung function tests	Completed	No publication found
NCT01750411	Asthma	Blood, lung function testing	Genetic influences on disease severity and the use of statistical modeling techniques to better understand disease phenotypes	Active, not recruiting	
NCT02721134	Sepsis	Blood	“New biomarkers” (somehow based on a LPS assay—not clear from the description)	Recruiting	
NCT03109288	Multiple sclerosis	Blood, tears, spinal fluid	“Biomarkers”	Recruiting	
NCT00942214	Multiple sclerosis	Blood	HLA-alleles, “biomarkers” not further specified	Completed	Partly in ref. ^135^
NCT01060410	Systemic lupus erythematosus	Blood	Genetic polymorphisms of drug metabolizing enzymes and pharmacokinetics of cyclophosphamide	Active, recruiting	
NCT03033095	Spondyloarthritis	Blood	Calcium-binding protein complex S100A8/A9, prealbumin, haptoglobin (Hapto), protéine C-réactive (CRP), α1 anti-trypsin, apolipoprotéinA1 (ApoA1), platelet factor 4 (PF4), S100A12 protein	Active, recruiting	
NCT00251017	Patients receiving vancomycin	Blood	Single nucleotide polymorphism (SNP) of OAT1, OAT3, and OCT2, plasma creatinine and vancomycin concentration	Completed	No publication found
NCT03015610	Pediatric gastroesophageal reflux and asthma	Blood, lung function testing	Effect of CYP2C19 and ABCB1 genes on pharmacokinetics of lansoprazole, questionnaires, lung function tests	Not yet open	
NCT00895271	Immunodeficiency and immunodysregulation	Skin samples	Skin samples to be transformed into pluripotent stem cells for gene-therapy approaches	Active, recruiting	
NCT02508584	Chronic mycoplasma hominis septic arthritis		Personalized M. Hominis anti IgG	Active, recruiting	
NCT01699893	Immune System Response in general	Blood, nasal swab, stool, skin biopsy	Not specified	Completed	See ref. ^3^
NCT02690285	Healthy volunteers, later targeted to patients with Pyruvate Dehydrogenase Complex Deficiency	Blood	Glutathione transferase zeta 1 (GSTZ1) haplotype status	Completed	
NCT02929745	Psoriasis	Blood, skin biopsies	Comparison of HLA-Cw6 positive/negative psoriasis skin lesions at the single cell level	Active, recruiting	

Search results obtained from Clinicaltrials.gov using different key words until 3 Aug 2017: “personalized” –>1404 studies; “precision” –>738 results; (“precision” OR “personalized”) AND “immunology” –>157 results; (“precision” OR “personalized”) and “allergy” –>162 results; (“precision” OR “personalized”) AND (“Immunology” OR “Inflammation”) –>201 results; (“precision” OR “personalized”) AND “HIV” –>76 results; “systems medicine” –>14 results; “systems biology” –>51 results

*COPD* chronic obstructive pulmonary disease, *LDL* low density lipoprotein, *LPS* lipopolysaccharides

^a^Information within quotation marks are directly cited from clinicaltrials.org

While some issues including the timely collection of time-series samples have been successfully exercised in many classical clinical trials,^16–19^ other challenges still exist in several areas, such as coordination, ethics approval, data protection, high-frequency or dense sampling, sample measurement/data generation, data management, integration and analysis. First, the coordination of complicated clinical trials to obtain multi-layer “Omics” data itself is already challenging. That is, to convince, to organize and to synchronize activities of various partners such as clinical partners, biobanking, cell sorting facilities, experimental laboratories, data managers, computational analysis groups, sample transportation logistics among different partners requires the involvement of high-level leadership. Second, many biological processes such as transcription and metabolism change very fast and almost all the biological processes and immune cell functions are under the regulation of circadian rhythms and other negative feedback-based mechanisms. We might have to take time-series samples with short intervals for some immune diseases according to the Nyquist-Shannon sampling theorem.^20^ Based on our experience in clinical trials, it requires completely different magnitudes of efforts and resources to perform time-series sampling with intervals in the order of years, months, days, hours or minutes. So far, most longitudinal cohorts largely sample from patients with an interval of years/months or in the best cases weeks or days, which still only entails routine efforts. Third, how to fully make use of same small quantities of patient samples or biopsies for a simultaneous measurement of different types of molecules and cells is also filled with challenges because different types of molecules might require very different procedures of sample preparation. Fourth, a huge challenge is the integration of resulting multi-layer “Omics” data sets with clinical data since we are fully aware how difficult to handle even just a single type of genome-scale data sets is. Fifth, what seems to be simple in the aspects of ethic authorization and patient data protection, in reality is highly challenging, which will be discussed in the later sections. Last but not least, we also need to overcome other complicated aspects, to name but a few, the burden of high financial costs of multi-“Omics” approaches, which is related to not only genome-scale measurement, but also a larger number of personnel required for dense time-series sampling, and to conquer the barriers of distinct expertize and standards of involved laboratories/computational groups (Table 2). Our ongoing study (NCT02931955) that only focuses on a model disease to establish a multi-layered time-series approach to understand its genetic and molecular characteristics has to overcome all the aforementioned issues. In the following paragraphs, we highlight some examples and discuss the potential future focuses of selected fields of preclinical and clinical research.

**Table 2:** Summary of key challenges and the potential solutions towards personalized immunology

Items	Key challenges	Potential solutions
1	Genome-scale or finer-scale analysis on “averaged” data of heterogeneous cell types from body fluids (e.g., blood or PBMC) or biopsies	Cell-type-specific and state-specific “Omics” analysis on sorted immune cells
2	“Averaged” results of heterogeneous individual immune cells	Single-cell “Omics”
3	Lack of disease progression and clinical-outcome predictive, prognostic and early-warning tipping-point biomarkers	Dense time-series “Omics” measurement and analysis along longitudinal studies
4	Lack of comprehensive profiling of various types of molecules	Multi-layer “Omics” and integrated experimental and computational analysis
5	Focus on our own human cells	Also with skin, lung, gut, and reproductive tract microbiome analysis
6	Lack of large effects of identified SNVs on the diseases or symptoms of interests	Selection of patients or subjects with more defined inclusion or exclusion criteria, e.g., removing those with comorbidity; combinatorial effects of higher number of SNVs with more powerful computers
7	Availability of research-focused genetic analysis tools	Clinics-orientated standardized genetic analysis tools with higher accuracy, stability and computational power
8	Only a small fraction of patients with up- or down-regulated biomarkers identified by group-wised approaches	Personalized expression perturbation profiles of each individual
9	Biomedical interpretation for biomedical researchers or clinicians using machine-learning based classification approaches not provided yet	Personalized expression perturbation profiles of each individual
10	Unreliability and irreproducibility in identified single or a panel of molecular biomarkers	Standardization in clinical sampling procedures, sample measurement, data management and analysis; Absolute quantification of biomarkers of interests using a large-number of “Omics” data sets as a reliable common reference; Personalized sample-specific network (SSN)
11	Relevant immune cells or molecules of interests often show nonlinear dynamic characteristics	Time-series space-state analysis
12	Instability of transcripts and metabolites	Proteomics-based analysis
13	Lack of information of immune cells about environmental exposome	Epigenomics-based analysis
14	Massive unstructured and unstandardized clinical data	Reliable and efficient text-mining tools
15	Lack of integration of prior knowledge on disease mechanisms with potential biomarkers	Establishment of molecular maps for different diseases.
16	Fragmented, unstandardized, unsecured, undigitized, unstructured, uncentralized, and ever-increasing big data	Dedicated big-data management platforms and shared national and international infrastructure with long-lasting update.
17	Classic informed consents (ICs) with defined duration and research purposes	Broad or dynamic ICs
18	Threat of patient data privacy due to wide usage of social-media or wearable-instruments derived clinical or behavior information	New anonymization and pseudonymisation approaches of patients’ identification
19	Group-wised approaches to assess efficacy and safety of candidate drugs	Separate evaluation of effects on individuals or subgroups of patients
20	High and long-lasting financial cost	To adjust and extend the current funding period framework for most agencies; Closely working with health insurance providers to differentially treat patient subgroups
21	One-cut pharmaceutical production pipelines	Multi-“Omics”-guided customized production pipelines

Literature citation is directly inserted through the main text due to a large-number of references.

### Autoimmune diseases—systemic lupus erythematosus (SLE)

SLE is an autoimmune disease, predominantly affecting young women, with a cutaneous, vascular and other auto-inflammatory manifestations. Especially kidney involvement in the form of nephritis determines the prognosis. As of today no single treatment is curative and there is enormous heterogeneity in both clinical and molecular patterns.^21^ Therefore, it is essential to stratify patients for improving personalized treatments. For this purpose, Banchereau et al. have first sampled blood from hundreds of pediatric SLE patients through a longitudinal cohort and analyzed the whole blood transcriptome. Following that, they have applied weighted gene co-expression network analysis approaches^22^ to identify patient-specific co-expressed modules that were best correlated to clinical traits over time. They then used clinical-relevant patient-specific modules to cluster and stratify patients into seven patient groups. Accordingly, they were also able to precisely stratify additional test patients into corresponding subgroups. These co-expression modules might be highly valuable to stratify patients into subgroups, which requires longitudinally sampling, however, that might be not accessible for most patients.

While this is an important step forward, this concept still needs to be translated into clinical practice so that a patient stratification like this can be used for the identification of a potential therapeutic or disease-monitoring approach. To further evaluate this potential, an integration of genomic, metabolomics and other layers of data is needed. Moreover, the information of the whole blood transcriptome as performed^21^ might still only partially reflect all the manifestations of SLE, and we might even overlook some important disease-specific or patient-specific changes that could been “averaged” out due to the fact that the given genes might be expressed in multiple types of immune cells.

In parallel, we could also stratify patients with SLE using protein information, such as autoantibodies. Budde et al. have recently developed a middle-scale assay with up to 86 antigens to detect diverse autoantibodies involved in various pathways.^23^ Using such an approach, they were able to separate SLE patients into five clusters. Such an autoantibody-based approach might also contribute to precise patient stratification, especially for autoimmune diseases. We believe that with the additional information on other layers of “Omics” data and the extension to a wider spectrum of autoantibodies, more precise stratification of SLE patients should be possible in the near future.

### Allergic diseases—allergen immunotherapy

Allergies affect >10% of most countries’ populations and are cause for significant secondary diseases and financial burden.^24^ Currently, the only curative option is allergen immunotherapy (IT).^25–27^ It works through complex immunological processes starting with mast cell and basophil desensitization that lead to changes in the T-cell compartment^28^ and finally modifications in B cells as well as mast cells, basophil, and eosinophil allergen response patterns.^25^

The component characterization of allergens^29^ has considerably improved molecular diagnostics of allergies, for example, the differentiation between double sensitization to bee and wasp venom and cross-sensitizations, which helps to establish tailored IT.^30,31^ Furthermore, with higher-resolution component analysis we were able to show that predominant IgE sensitization to Api m 10 in individual patients might be a good predictive marker for the failure in IT treating honey bee venom allergy.^32^ However, success of the therapy cannot be predicted by classical approaches including measuring specific IgE or IgG4, skin testing or basophil activation tests.^27,33^

In order to better predict the success of IT, Ryan et al.^27^ have analyzed the TCR repertoire and expression of a preselected list of 24 genes of single CD4+ T-lymphocytes of peanut allergic patients undergoing oral IT, and of healthy controls. Patients’ CD4+ T-cells could be clustered into seven groups that showed complex phenotypic changes in CD4+ lymphocytes over the course of oral IT, and distinct temporal changes were especially observed in the antigen-specific CD4+ lymphocytes. Patients who successfully passed an oral double-blind placebo controlled food challenge demonstrated a shift towards a “tolerant” Th2 phenotype only 3 months after the induction of IT.^27^

Although such a wide-spectrum analysis is already quite powerful in predicting the success of IT, this information only represents a small fraction of all the manifestations that truly reflect the patients’ immunological profiles. Systemic level deep phenotyping of various relevant immune subsets and profiling genome-scale expression or concentration levels of transcripts, proteins and metabolites of each type of relevant immune cells should provide much more precise and unbiased molecular characterization and stratification in combination with classical clinical information, which for instance is being pursued in the clinical trial initiated by us (NCT02931955, Table 1).

The IT itself will be the focus of future research and become more precise and tailored to individuals in the next years. As an initiative, the recombinant allergens produced for diagnostic purposes provide an interesting option for future tailored personal IT and might replace todays’ crude whole venom/allergen extracts that might vary in their composition.^34^ This might help to reduce the number of patients suffering from side effects of the therapy as it affects up to 40% of patients undergoing subcutaneous IT,^35^ which need to be lowered to ensure patient adherence, with the assistance of “Omics”-based stratification.

### Infectious diseases

Infectious diseases are a major cause of morbidity and death worldwide, fostered by diagnosis sub-efficiency and poor access to treatment, especially in developing countries, whereas rising antimicrobial resistance appears as a main challenge to global public health. Monitoring and cure of infectious diseases is particularly challenging from the view of personalized medicine, since it first requires comprehensive understanding of both host and pathogen’s individual features prior to elucidating how their interactions determine the outcomes of infection in different patients. Transcriptomic studies of patients’ peripheral blood mononuclear cells (PBMCs), CD4+, and CD8+ T cells during the course of infection were investigated to identify genes associated to survival, exhaustion or memory phenotype.^36,37^ However, these studies did not assign predictive biomarker values to the identified lists of genes.

Inter-individual and intra-individual variability in adaptive and innate immunity, as well as age, gender, ethnicity, microbiome, environmental factors (also known as exposome) or existing diseases influence the overall responses to antigens. Systemic studies^38^ of these parameters with regards to the response to vaccines will probably help to predict vaccination outcomes, by identifying molecular signatures induced after vaccination, unraveling their biological causative mechanisms, and assessing their predictive value towards a responder status.^39^ In this regard, systems vaccinology could allow for identification of early personalized molecular signatures linking to vaccination, which could monitor or predict the efficacy of a particular vaccination strategy or could help identify patients at risk for systemic reactions after vaccination.^40^ Through a transcriptomics analysis, Fourati and coauthors identified an age-related signature and a 15-gene signature predictive of vaccine hypo-responsiveness to hepatitis B virus surface antigen in naïve older adults.^41^ Possibly due to the fact that the transcriptomic analysis was based on whole blood samples, their predictive power was quite limited. These examples show that the first shots applying “personalized immunology”-like approaches to infectious diseases not only enable researchers to fully document the changes occurring in the host after pathogen transmission or its vaccine counterpart, but also provide a major tool to predict the outcomes of infection or vaccination before it occurs.

## Major challenges towards personalized immunology

### From whole tissue to cell-type-specific and state-specific “Omics”

So far, most of genome-wide studies report on “averaged” results of different immune subpopulations, e.g., from PBMCs or even whole blood or other body fluids or biopsies (Table 2). Those “averaged” results of heterogeneous cell types prohibit further in-detail molecular profiling and functional evaluation of individual cell types and their interactions in complex diseases.^42^ Although advanced in-silico cell deconvolution approaches have been recently developed to extract cell-type specific information from whole tissues,^43^ they are suffering from serious limitations. For instance, it can mainly fruitfully analyze groups of patients vs. healthy controls, which apparently contrasts to the requirements of personalized medicine. Therefore, identifying key molecular and cellular players in personalized immunology can only be successful if separated specific cell subpopulations will be assessed regarding their “Omics” data on a more and more precise level, e.g., starting from analyzing CD4+ T cells and then further separating them into their subtypes e.g., regulatory T cells (Tregs), Th1, Th2, Th17 and others. One step further, many diseases are mainly characterized by dysfunction in particular subsets of immune cells at particular activation or other functional states while the development of those immune cells is intact. For instance, the tumor infiltrating CD8+ T cells predominantly display an exhausted phenotype (state) and the magnitude of reinvigoration of peripheral exhausted T cells in relation to pretreatment tumor burden determines clinical outcomes of individual patients.^44^ Unless we perform “Omics” analysis on the very sorted subsets of immune cells at the given states, including but not limited to, memory vs. naïve, resting vs. stimulated, exhausted vs. non-exhausted T cells^45^ (Fig. 1, Step 4–5), we could not unbiasedly figure out the myriad facts whether and how particular subsets of immune cells at the given states could contribute to or predict the clinical outcomes of specific patients following treatment. This demand to more deeply investigate cell-type-specific and state-specific “Omics” analysis (Fig. 1, Step 4–5) is further complicated by tissue-resident immune cells, which might display completely different molecular patterns between different tissues, as do human memory T cells from bone marrow and PBMC for example.^46^ The tissue-resident immune cells might also contribute to the pathogenesis of many diseases. The state-of-the-art multichannel fluorescence activated cell sorting (FACS) followed by “Omics” analysis permits such an aforementioned assessment.

### Single-cell (sc) “Omics”

Cellular type-based “averaged” approaches have made major contributions in understanding molecular networks and functions of cells. However, no single cells even for the same cell type are identical and we do not know what the “averaged” values exactly mean (Table 2). Such distinction among different individual cells could be caused by mutation, stochastic variation or environmental perturbations.^47, 48^ The difference might be reflected on various molecular levels (DNA, coding and noncoding transcription, translation, metabolism, epigenetic modifications and other levels). The molecular heterogeneity among individual cells might eventually cause functional heterogeneity.^49^ Such molecular heterogeneity might also be attributable to the various activation stages, which might be particularly true for immune cells.

Based on recent breakthroughs (Fig. 1, Step 6) in sc-transcriptomics,^50^ sc-proteomics^51^ and even simultaneous measurement of epitope and transcriptomics in single cells,^52^ we are now in a unique position to characterize the molecular and functional heterogeneity of rare cell populations. Significant progress has already been made in sc-studies to better characterize individual tumor cell heterogeneity in cancer and brain as well as to illustrate heterogeneity of certain immune cells, e.g., macrophages^53^ and dendritic cells.^54^

Furthermore, recent advances in epigenetics have allowed us to perform sc-epigenomic analysis^55,56^ in various types of cells, such as embryos,^57^ primary lung adenocarcinomas, fibroblasts,^58^ and hepatocytes.^59^ More excitingly, advanced techniques have also been developed for sc-metabolomics analysis, especially for analyzing those circulating cancer cells that lead to metastasis.^60^ However, these approaches have yet to be comprehensively applied to the rare populations of immune cells, for instance, including Tregs,^61^ natural killer cells, innate lymphoid cells, and others. In further steps, these methods could be combined with imaging techniques, e.g., ImageStream that combines the power of flow cytometry and microscope, to further enhance the knowledge on immune cell function, protein–protein interaction and cell–cell interaction.^42^ Applying these multi-omics approaches to a wider setting relies on maximization of coverage, accuracy, and reproducibility in the coming years and will allow more precise predictions from geno-type to endo-type to pheno-type.^42,56^

The next challenging question is to which degree we can apply single-cell based analysis to diagnostic or prognostic purposes in real clinical settings since the heterogeneity among individual cells might be even higher than the heterogeneity among individual patients. One can envision that the heterogeneity degree for a given cell type might increase or decrease in patients with certain diseases. If this is the case, the heterogeneity degree or the frequencies or clusters of finely-characterized cell subsets^53^ can be used as biomarkers. The number of required single cells to provide reliable insights into the heterogeneity of gene expression, methylation or metabolism has to be balanced between the analysis cost and required statistical power. With the decreasing cost in sequencing techniques, we shall have more power to identify reliable diagnostic and prognostic markers from isolated single immune cells for immune-related diseases. Although a few clinical trials are ongoing or were just finished (e.g., NCT02929745 and others, Table 1), no work has so far reported the usage of sc-transcriptome to identify diagnostic or treatment-outcome predictive biomarkers in clinical studies, which will be foreseen in the coming years.

### Longitudinal studies and dynamic analysis

**Fig. 2:**
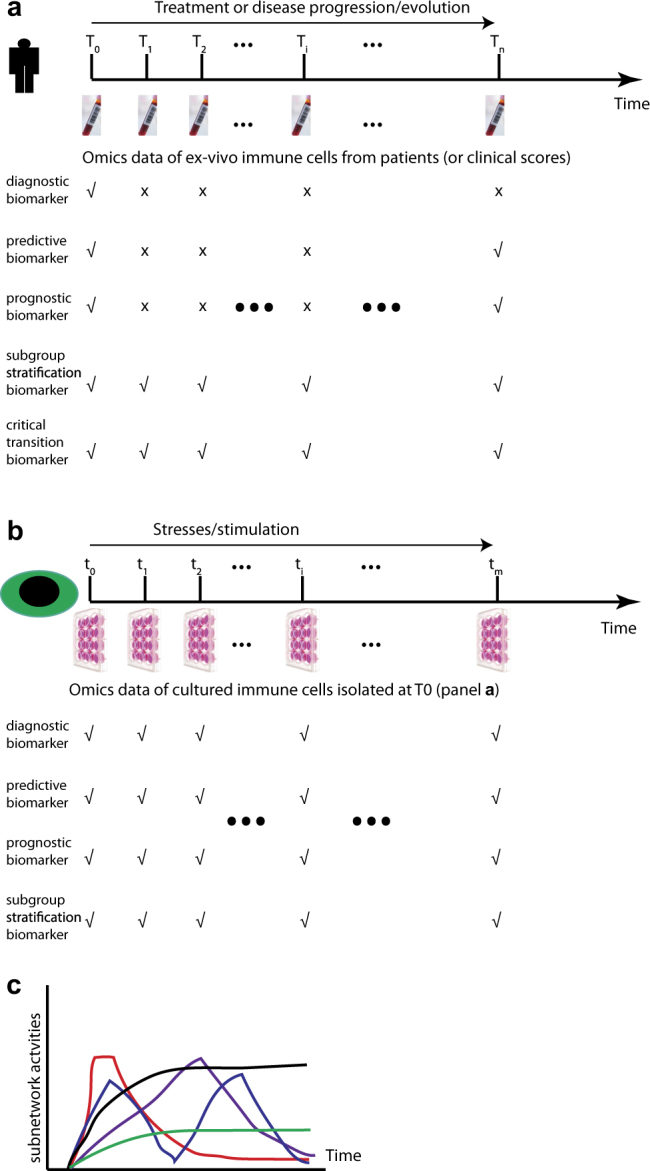
Longitudinal studies and dynamic measurement are critical for discovering various types of biomarkers. **a** Longitudinal follow-up of individual patients with multilayer “Omics” analysis is essential for identifying different types of biomarkers. The check marker at the given time point indicates the necessary “Omics” measurement and clinical assessment for revealing the given type of biomarker while the cross symbol indicates an unnecessary involvement at the given time point for the given type of biomarker. **b** Time-series “Omics” analysis of the cultured isolated immune cells from the first visit (T_0_ at panel **a**) following certain stimulation or stresses will also be able to help extract various types of biomarkers. **c** Various types of dynamic patterns of different pathways or modules or subnetworks of the given relevant type of immune cells isolated from PBMC or other tissues of individual patients might be valuable for patient subgroup stratification. Subnetwork activities at the given time can be defined either by the expression levels of the co-expressed genes, or by the expression levels of the effector genes (such as cytokines) or any other readouts which could define the activities or outputs of the given pathway or subnetwork or module

While the concept of prospective longitudinal follow-up is a key element of clinical studies to help identify prospective risk factors, prognostic and treatment-efficacy biomarkers for diseases (Fig. 2), this still needs to be implemented in modern omics-based research (Table 2). It might be supported by establishing a lineage-tracing tree retrospectively as demonstrated regarding acquisition of mutations in cancer cells.^56^ Moreover, establishing longitudinal cohort studies is essential for detecting predictive biomarkers from “Omics” analysis, e.g., the critical transition early-warning biomarkers^62^ (Fig. 2a), before the appearance of apparent clinical symptoms,^63^ when it might be often too late for an effective therapy or cure to many chronic diseases.^64^ Identification of those early-warning biomarkers could profoundly help us to decide when and how to apply preventative treatments to many chronic diseases which are incurable so far (Table 2). This will then need to be applied to a prospective approach in a personalized manner.

Heterogeneity in patients is reflected not only in the expression levels of coding and non-coding genes and proteins, concentration of metabolites, activity of signaling transduction proteins, or combination of them, but also in the resulting molecular interaction networks, especially in the dynamic response patterns and functional output of the networks following physiological or pathological stress. However, all the “static” biomarkers lack of information on any underlying molecular mechanism, which, at least partially, explains the current conundrum why most of biomarkers are not necessarily suitable therapeutic or prognostic targets. In order to provide mechanism-based biomarkers and the following stratification of patients, the pioneers, e.g., Kholodenko and his colleagues have already started to explore novel prognostic markers by simulating the dynamic output of relevant signaling transduction pathways based on transcription expression levels measured in individual tumor patients.^65,66^ Strikingly, they have already successfully shown that the various output indexes, such as the maximal amplitude (A), the activation threshold (K50), and the Hill exponent (H) of the Jun N-terminal kinase signaling responses of tumor cells (Fig. 1, Step 6), can be used to predict poor or favorable survival of patients with neuroblastoma.^65^

In the immune systems, the degree of heterogeneity is even more complex not only regarding numerous types of immune cells, but also various states of immune cells, and even transient cell types with bidirectional-convertible plasticity. The heterogeneity degree is even further complicated by the interactions of such a broad range of immune cells. On a molecular level, it has already been demonstrated that the gene modules that were organized based on the transcript expression patterns in the peripheral blood of elderly individuals can also be used to stratify those individuals into two completely different clinical and immunological states.^67^ More detailed work is expected to characterize various types of molecules, not only in signaling transduction dynamics (Fig. 1, Step 6), but also in dynamic patterns of gene expression and epigenetic modification (Table 2) of the particular isolated immune subsets that should be able to provide much better predictive and stratification power (Fig. 2b, c). To the best of our knowledge, this promising direction is unfortunately still missing in immunology.

With the possibility to obtain time-series large-scale measurements on various types of molecules and immune cells of patients within well-designed longitudinal cohorts, we will be in a unique powerful position. We will be able not only to describe the molecular and cellular dynamic patterns^68^ (Fig. 2a–c), but also to predict possible upcoming immune state changes of the patients. The well-established space-state models^69–71^ that are widely used in various fields such as finance, ecology, population dynamics, and weather forecast should be adaptable and utilizable in immune state prediction using time-series data sets of longitudinal cohorts (Table 2). There are at least three ways to predict immune disease states, also known as attractors in mathematics.^72,73^ One can build state-space models by using dynamic measurements of various layers of molecules within each type of relevant immune cells, or using dynamic measurements of frequencies and functional markers, such as activating or inhibiting receptors or cytokines/chemokines, or cytotoxic mediators of each type of relevant immune cells. Ideally, one might need to combine both dynamic patterns of various layers of molecules and of various types of immune cells since with the known activating or inhibiting receptors alone we cannot reveal all the functional aspects and response potential of the given immune cells. Only in this way, one could make full use of the precious time-series large-scale data sets to predict future immune disorders or disease progression^74–76^ while keeping in mind the apparent drawbacks^77^ of the current state-space models, such as inability to predict long-term trajectories.^78^

### Microbiome analysis

There is a clear link between the human gut microbiome and the development of immune-related diseases, e.g., inflammatory bowel disease and allergies.^79,80^ In general, higher diversity of one individual’s microbiota is correlated with a reduced risk for the development of asthma and other allergies and can to some degree even predict the development of allergies.^81,82^ Using a machine-learning approach, microbiome data were used (Fig. 1, Step 3), for example, to stratify patients with irritable bowel syndrome successfully into subgroups.^83^ Tejdo et al. were able to correctly identify patients with Crohn’s disease based on their fecal microbiota composition.^84^ While the lung historically was considered microbe-free, knowledge on the human lung microbiome is steadily increasing over the last years, thanks to the advantages in next-generation sequencing based techniques.^85^ The human microbiome data set (http://hmpdacc.org) shows that an abundance of bacterial species are present in the lung and this colonization is different in asthmatic individuals compared to healthy persons.^86,87^ Furthermore, a reduction in microbiota variety after antibiotic treatment is clinically correlated with a reduced bronchial hyper-responsiveness.^87,88^ This hints at an interplay between lung microbiome and asthma development. Reduced diversity of skin microbiota was also found in the patients with atopic dermatitis^89,90^ or psoriasis.^91^ These studies show that skin, gut, and lung microbiome could potentially be used as diagnostic and treatment-efficacy biomarkers in the context of many other immune mediated diseases. Furthermore, reported inter-individual differences in the gene content of human gut bacterial species^92^ might designate the necessity to develop approaches to use the additional layers of information on patient-specific microbiome to precisely stratify patient’s subgroups (Table 2). Ultimately, this should not only cover diagnostic or prognostic aspects, but move towards therapeutic approaches as well.^79^

### Personalized network and computational analysis

Following the generation of large-scale “Omics” data, the next challenging step is to computationally analyze these data to precisely stratify patients into subgroups based on various sets of biomarkers at different molecular layers in combination with frequencies of immune cell subsets, clinical information, and epidemiological data. Significant efforts have already been put to identify genetic variants that are associated with certain traits, for instance, identification of robust association between single nucleotide variant (SNV) with single traits or complex diseases.^93^ Although many computational algorithms have been developed for this purpose, unfortunately, most of the identified SNVs have very small effects on the given traits or diseases. Not mentioning any other type of “Omics” data, with unprecedented amount of genomic sequencing data following the announcement of the precision medicine initiative in many countries, we definitely need more powerful alignment and assembly algorithms.^93^ Those approaches are critical to empower us to perform more accurate variant call and whole-genome sequencing analysis^94^ to identify patient-specific variants that contribute to the pathogenesis of a given disease in an individual patient. In this context, clinics-orientated vs. research-focused genetic analysis tools that require higher accuracy, stability, and computational power/speed are still underdeveloped.

Traditionally, clinicians have already utilized the information of metabolite concentration, e.g., serum glucose levels for diagnostic purposes (Fig. 1, Step 1). More recently, researchers have already developed and applied various bioinformatics tools to utilize large-scale data, i.e., metabolomics data to seek biomarkers^95^ (Fig. 1, Step 3). For instance, researchers have already commenced to use metabolomics data as cancer early predictive, diagnostic and therapeutic treatment response biomarkers.^96^ Researchers have also made use of metabolomics data as biomarkers for various autoimmune diseases, such as Crohn’s disease^97^ and recently also rheumatic diseases.^98^ Since metabolites are in one of the effector layers of cellular functions, there are certain advantages to utilize metabolomic data as biomarkers. Although technically more challenging compared to mRNA analyses, the intracellular metabolome of a specific type of immune cells should provide not only novel insights into immune-metabolism,^99,100^ but also more precise cell-type-specific biomarkers.

Current efforts to identify biomarkers and stratify patient subgroups are mainly put on transcriptomic data that are obtained either from probe-preselected microarray measurements or unbiased RNA-sequencing based techniques. Many statistical approaches were applied to identify differentially expressed biomarkers (Fig. 1, Steps 3 and 7). However, the biomarkers discovered based on those group-wised approaches are up- or down-regulated only in a small fraction of patient groups compared with healthy controls.^101^ Furthermore, due to the limited number of training samples, bioinformaticians often generate a long list of differentially expressed biomarkers. However, to successfully apply this to the real life of clinical settings, the number of biomarkers in the panels should be limited. Machine learning approaches, such as Bayesian classifiers and support vector machine have been successfully applied to various studies to stratify patient groups.^102^ However, machine learning approaches are black boxes for most of biologists and clinicians that can hardly provide biomedical interpretation (Fig. 1, Step 7). To address this limitation as well as the huge heterogeneity among different patients even for the same diseases, Barabasi and his group have recently developed an approach to utilize the personalized expression perturbation profiles of each individual based on transcriptomic measurement as the barcode for each individual for the studied disease^101^ (Table 2). They found that the fraction of genes from the identified disease module perturbed in an individual subject can accurately predict the status of the given individual (Fig. 1, Step 7). Compared with the machine learning approaches, this combinatorial model could be more explicit. However, like any other method, this approach has also its own limitations, such as one needs to first decide and optimize the threshold to define whether the genes are perturbed or not and one is still not sure whether this method is beneficial for a further stratification of the patients with the same diseases into subsidiary groups.

Unreliability and irreproducibility in identified single or a panel of molecular biomarkers has already caught enormous concerns by both academia and pharmaceutical companies.^103,104^ Such irreproducibility could be caused by either technical issues or intrinsic biological variance itself or a combination of both factors. Ubiquitous existence of irreproducible biomedical results could also be attributable to a lack of standardization in error-prone affairs, such as sample preparation, sample measurement,^105^ data analysis^106^ or the combination of all the steps (Table 2). For instance, regarding clinical sample measurement, worldwide standardization of a diagnostic test is the development of the international normalized ratio (INR) to measure the extrinsic pathway of coagulation. Initially the prothrombin time was used but varied greatly by the various laboratories providing the results. An international sensitivity index of the reagents used is thus determined by testing an international reference tissue factor (ISI).^107^ A similar approach might be extended and adapted to different “Omics” measurements.

In the meantime, to recapture the reported expression or concentration level changes of individual molecules such as mRNAs, proteins or metabolites are tricky, not only due to their dynamic characteristics of nonlinearity, such as circadian rhythm-driven fluctuations and technical challenges, but also due to environmental, nutritional or even emotional influences. In order to mitigate technical challenges that are partially responsible for data irreproducibility in genome-scale mRNA measurement, Seita et al. have used a large number of microarray data sets as a common reference to estimate the absolute expression values of each gene of interest^108^ (Table 2). In this way, one could at least have opportunities to quantify the biomarkers of interest, to more precisely compare them with the counterparts of control groups in order to support clinical decision making. Otherwise, due to the variability caused by technical challenges alone, one cannot determine whether the given genes or other biomarkers have been really up-regulated or down-regulated in the given patients and therefore might conclude a wrong diagnosis or even suggest an inappropriate treatment. More recently, Chen and his colleagues have proposed to use networks that are supposed to be more resistant to both technical and biological variances, instead of single-molecular biomarkers^109^ (Table 2). They identified personalized SSN biomarkers by calculating the differential correlation networks between the reference groups and that target group combining the reference groups with the single sample from the patient of interests using transcriptomic data (Fig. 1, Step 7). They have successfully demonstrated a very high performance (>98% accuracy) when classifying tumor samples. Importantly, using their approaches they identified that most of the hub genes in the differential correlation networks are non-differentially expressed and they have experimentally validated their functional importance in drug resistance. Coincidently, the discovery of non-differentially expressed key genes has also been demonstrated by our previous work.^110^ Those network biomarkers represent a novel future direction which possibly renews the definition of traditional biomarkers and could be widely used in personalized medicine including personalized immunology, since it requires only a single sample for each individual. However, one still needs to be cautious when it is applied to immune diseases due to extra dynamic characteristics of various types of immune cells during the disease progression and even just due to biological circadian rhythm.^111^ Following activation, if the dynamic characteristics of the relevant immune cells or of the molecules of interests show nonlinearity, which might be the case in many situations, a simplified network-based stratification approach will not be so efficient anymore.

Due to the relative stability of proteins, proteomics-based computational approaches have been developed to identify more robust biomarkers, especially in the field of oncology.^112,113^ A growing body of evidence shows that many diseases are developed following exposure of various types of acute or chronic environmental factors (Fig. 1, Step 3), it therefore makes sense to assess their effects on the epigenetic states of different cell types including immune cells. However so far, few methods have been established to identify epigenetic biomarkers.^114,115^ In order to further improve the accuracy and confidence levels, which is extremely important in the clinical setting, integration of various layers of molecules and clinical information is essential to identify reliable multilayer biomarkers and to more precisely stratify patients into appropriate groups for personalized treatment (Fig. 1, Step 3). For instance, in clinical settings, diagnostic false negative rates might be more harmful than false positive rates in many type of diseases, and another way around for many others. Conversely, in the research field, the two types of rates might be equally treated. Last but not least, we also need to develop reliable and fast text-mining tools to extract clinical information from unstructured and unstandardized clinical data^116^ (Table 2). This aspect is also not trivial since the clinical data in many hospitals or clinical centers still do not make the structured and standardized prescription or description compulsory while the volume of such data is exponentially increasing.

With the development and accumulation of our understanding in molecular mechanisms of a wide spectrum of diseases, researchers have already initiated concepts and approaches to reconstruct, visualize and analyze diseases molecular maps, such as the Alzheimer’s disease map and the Parkinson’s disease map.^117,118^ We are convinced that with the support of prior knowledge, i.e., disease maps (Table 2), we should be able to not only more precisely stratify patients according to the particular pathways/subnetworks that were detected in the given individual patients, but also in a much more intuitive manner to support clinicians to better understand the pathological mechanisms, to make decisions and to differentially treat patients. However, in contrast to the neuron-focused maps in neurodegenerative diseases, different types of immune cells with different dynamic stages that are often involved in immune-related disorders, make the development of such disease maps more challenging. The fundamental limitation in stratifying patients using disease maps is whether we could identify any novel subgroup of the patients of interests based on prior knowledge, which might be not so critical anymore if the diverse mechanisms underlying the given disease have been well understood.

So far, most of network analysis approaches are based on single-layer “Omics” data. However, our molecular and cellular networks are in fact composed of various types of bulk of molecules, such as genomic DNA, mitochondrial DNA, coding mRNA, noncoding mRNA, proteins, metabolites and epigenetic modifications. Furthermore, our bodies consist of not only our own cells, also billions of symbiotic microorganisms that could be beneficial or pathologic to our immune systems (see above). Therefore, it is urgent for us to integrate various types/layers of “Omics” and clinical data^119,120^ to build more comprehensive multi-scale and multi-layer network models to stratify patient subgroups for the complex diseases of interest (Fig. 1, Step 3). To reach that aim is still a long way to go due to the various reasons such as high financial cost, lack of appropriate computational approaches, lack of common understanding of joint expertise, and others.

### Big-data management

To be more efficient, compatible and secure, all medical and large-scale “Omics” data sets of patients, whatever the diseases they are concerning, will have to be digitized, integrated, structured, centralized, secured, and standardized^121^ (in acronym, “DISCSS”, Table 2). Not only is standardization in experimental and clinical sampling procedures required, but also standardization in big data formatting, description, repository, analysis, integration, and sharing is vital to the success of personalized immunology (Table 2). Furthermore, high-resolution medical imaging data, behavior and symptom/phenotypic data derived from social media^122^ and wearable instruments and smart phones^123^ will generate unprecedented ever-increasing volume of clinically related data. For this purpose, dedicated integrated large-scale biomedical data management platforms,^124^ such as TranSMART,^125,126^ FAIRDOM^127^ and others are badly needed for diverse clinical or preclinical studies. International or national shared infrastructure on big-data storage, analysis and training with the highest standards to maximize the value of biomedical metadata, such as European collaboration to handle data in life science, also well-known as ELIXIR^128^ and BD2K (Big Data to Knowledge) initiative^16^ need to be further developed and popularized across the world. This meets the e-health concept, at least, by the European Commission within the frame of the H2020 initiative (https://ec.europa.eu/digital-single-market/en/policies/ehealth), shifting from physician-centered to patient-centered healthcare. Compliance with legal and regulatory aspects (General Data Protection Regulation for example) might prevent or impair this cross-border e-health concept, which is extremely important for Europe where mobility of workers across different countries is becoming routine. Centralized patient files with the explosive growth of molecular “Omics” data volumes will require long-lasting update to technological capacities, such as data storage space on servers, better and faster compression/decompression algorithms and user-friendly accessibility for physicians. General practitioners might not be able to afford costs, which means that patients’ files would be accessible from larger healthcare centers only. Hopefully, the health infrastructure tomorrow should be transformed by, and eventually support the implementation of personalized medicine.^129^

### Other miscellaneous challenges in implementation of personalized immunology

Besides purely scientific and clinical aspects, implementation of personalized immunology will imply the compliance of legal, regulatory, social, and technical issues that will also be briefly addressed here.

#### Ethic and regulatory issues

Through “Omics” technologies coupled to other systems biology approaches, huge amounts of personal health data sets will be generated and shared. According to current legislations on data privacy, a fully informed consent (IC) of the patients is required for data processing within the frame of duration-defined specific purposes. The advent of personalized medicine challenges this concept of IC, since it clearly implies secondary data processing or re-processing according to the FAIR principles (Findable, Accessible, Interoperable, and Reusable) of the large-scale data management,^106^ which is now required or at least recommended by NIH or EU funding agencies for the submission of new proposals related to generation or analysis of big biomedical data. Similarly, the discovery of predictive markers might allow the researchers/clinicians to be aware of unavoidable future diseases or debilitations. However, it remains unclear whether according to the classic ICs given to analyze data in the context of a particular disease, the patient should be informed of newly discovered risks of suffering from other diseases that have not been initially screened. Possibly, one should go for broad or dynamic ICs, which is however either too labor-tedious or still not well-accepted yet.^130^ Since the usage of freely-available social media-derived clinical information is ever increasing, privacy protection and anonymization of patients’ identification^131,132^ might become impossible in the near future^133^ (Table 2). How will the two aspects, i.e., patient data privacy, data sharing and ever-expanding possible secondary-usage purposes be reconciled?

Clinical trials to assess the efficacy and safety of candidate drugs are currently still mainly based on the group-wised comparisons, i.e., between the drug treated and placebo given groups (Table 2). This will still need to be addressed when individual patients will be treated with personalized drugs or vaccines or a combination of therapies. New guidelines on how to evaluate drug efficacies and safeties will need to be developed in the era of personalized medicine.

#### Cost issues

Identification of biomarkers requires the in-depth “Omics” characterization and stratification of population cohorts with sufficient power of statistics. These potential biomarkers will then need to be validated and correlated in different patient subpopulations, according to multiple parameters such as sex, age, ethnicity, and others.^134^ The implementation of personalized immunology should result in a reduced social and financial burden through fine-tuned patient stratification into some subgroups requiring simple and relatively cheap treatment, while others demanding much more complicated and expensive therapeutic alternatives (Table 2). Given the huge funding required to achieve this goal, clear criteria will have to be set to decide which diseases are worthy of these efforts. Most likely, the decision will be made based on the prevalence of a disease worldwide, or on the potential return of financial investment. However, since personalized immunology emphasizes personal molecular characteristics, rare diseases might pop up eventually as one of the focuses, which will further complicate these intuitive considerations in cost. Since longitudinal cohort studies are vital for simultaneously discovering various types of personal biomarkers, the current funding framework in many countries, which often only lasts 3–5 years, should be adjusted as well. Last but not least, it will be also challenging to adapt the current one-cut pharmaceutical production pipelines to the ones meeting the production requirements of personalized treatments (Table 2), such as personal vaccines, which might alter the entire concept of current manufacturing flows and apparently, at least the short-term return of investment.

## Concluding remarks

As outlined above, we discuss the multiple challenges and propose a roadmap, not only in scientific and clinical aspects, but also in big-data management, legal and regulatory sides towards personalized immunology. Among others, multilayer “Omics” analysis along longitudinal cohort studies are desperately required to simultaneously obtain various types of reliable biomarkers. Currently, approaches developed for patients’ subgroup stratification seem to be quite advanced relative to personalized therapies. One of the essential barriers that should be overcome in the near future is to translate patients’ subgroup stratification to personalized treatment. Currently, our success still mainly binds with patient stratification. Further developing both clinically applicable measurement and computational analysis approaches in personalized medicine is an incredible opportunity to increase global health, provided that investment returns are assessed not only in terms of financial profit, but also in terms of patient well-being. In other words, we should give ourselves a toolset to allow personalized immunology to be an advance for the sake of health and wealth.

### Data availability

No data sets were generated or analyzed during the current study.
