# Author Correction: A roadmap towards personalized immunology

**DOI:** 10.1038/s41540-025-00553-7

**Published:** 2025-07-12

**Authors:** Sylvie Delhalle, Sebastian F. N. Bode, Rudi Balling, Markus Ollert, Feng Q. HeFeng

**Affiliations:** 1 Department of Infection and Immunity, Luxembourg Institute of Health (LIH), 29, rue Henri Koch, 4354 Esch-sur-Alzette, Luxembourg; 2 Center for Pediatrics—Department of General Pediatrics, Adolescent Medicine, and Neonatology, Medical Center, Faculty of Medicine, University of Freiburg, Mathildenstrasse 1, 79106 Freiburg, Germany; 3 Luxembourg Centre for Systems Biomedicine (LCSB), University of Luxembourg, Campus Belval, 6, Avenue du Swing, 4367 Belvaux, Luxembourg; 4 Department of Dermatology and Allergy Center, Odense Research Center for Anaphylaxis (ORCA), University of Southern Denmark, 5000 Odense C, Denmark

Correction to: *npj Systems Biology and Applications* 10.1038/s41540-017-0045-9, published online 06 February 2018

In this article, the author name Feng Q. HeFeng was incorrectly written as Feng Q. He. The original article has been corrected.

